# Genetic Testing Distinguishes Multiple Chondroid Chordomas with Neuraxial Bone Metastases from Multicentric Tumors

**DOI:** 10.1155/2020/8877722

**Published:** 2020-11-28

**Authors:** Hiroshi Kobayashi, Masahiro Shin, Naohiro Makise, Aya Shinozaki-Ushiku, Masachika Ikegami, Yuki Taniguchi, Yusuke Shinoda, Shinji Kohsaka, Tetsuo Ushiku, Katsutoshi Oda, Kiyoshi Miyagawa, Hiroyuki Aburatani, Hiroyuki Mano, Sakae Tanaka

**Affiliations:** ^1^Department of Orthopaedic Surgery, Graduate School of Medicine, The University of Tokyo, Tokyo, Japan; ^2^Department of Neurosurgery, Graduate School of Medicine, The University of Tokyo, Tokyo, Japan; ^3^Department of Pathology, Graduate School of Medicine, The University of Tokyo, Tokyo, Japan; ^4^Division of Cellular Signaling, National Cancer Center Research Institute, Tokyo, Japan; ^5^Division of Integrative Genomics, The University of Tokyo, Tokyo, Japan; ^6^Laboratory of Molecular Radiology, Center for Disease Biology and Integrative Medicine, The University of Tokyo, Tokyo, Japan; ^7^Genome Science Division, Research Center for Advanced Science and Technology, The University of Tokyo, Tokyo, Japan

## Abstract

**Background:**

Chordomas are rare malignant bone tumors preferentially forming in neuraxial bones. Chondroid chordoma is a subtype of chordoma. Chordomas reportedly present as synchronous multiple lesions upon initial diagnosis. However, it remains unknown whether these lesions are multicentric or metastatic multiple chordoma tumors. *Case Presentation*. Here, we present the case of a 57-year-old woman with multiple chordomas at the clivus, C6, and T12 upon initial presentation. Sequential surgeries and radiotherapy were performed for these lesions, and postoperative histological diagnosis revealed that all lesions were chondroid chordomas. Next-generation sequencing revealed that these lesions harbored a common somatic mutation in epidermal growth factor receptor (*EGFR*), c.3617A>C, which is not considered a pathogenic chordoma mutation, thus indicating that these lesions were not multicentric but rather multiple metastatic tumors. Subsequent multiple metastases to the lung and appendicular and axial bones were detected 15 months after the initial surgery. Recurrent lesions at the clivus progressed despite EGFR-targeted therapy, surgery, and radiotherapy.

**Conclusion:**

The present evidence indicates that multiple chordomas in this case were caused by multiple metastases rather than multicentric lesions. Multiple presentations of chordoma imply systemic dissemination of tumor cells, and novel efficient systemic therapy is required to treat this disease.

## 1. Introduction

Chordomas are rare malignant bone tumors, arising predominantly at the axial skeleton. Consequently, they are believed to originate from remnants of the embryonal notochord [1]. Chordoma incidence rates were reported to be 0.18 to 0.84 per million people annually, and there are differences between races [2]. As for the anatomical distribution of chordoma, the sacrococcygeal region and skull base are affected in 26–32% and 29–45% of cases, respectively, and the mobile spine can also be affected. Chordoma is a locally aggressive tumor, and the reported local recurrence after surgery is 40–70% [3, 4]. Furthermore, chordoma tends to metastasize at rates of 18–30%, mainly to the lungs and bones as well as other organs [5, 6]. Chondroid chordoma is one type of chordoma, accounting for 14% of all chordomas. Chondroid chordoma is reported to have a better prognosis than conventional chordoma [7].

Multicentric chordomas with synchronous multiple presentations at first diagnosis have been rarely reported [8–10]. Methods for distinction between multicentric or metastatic multiple chordoma tumors have not yet been established.

Herein, we describe a case of multiple chondroid chordomas arising at the clivus, cervical vertebra, and thoracic vertebra. These lesions shared a common somatic mutation of the epidermal growth factor receptor (EGFR). This is the first report to describe the successful use of genetic testing to reveal that the multiple chordomas were not multicentric, but metastatic.

## 2. Case Presentation

**Figure 1:**
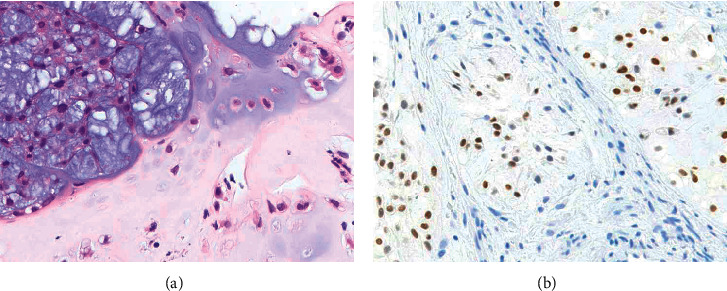
Histopathology of the tumor resected from the skull base of the patient. (a) Hematoxylin and eosin and (b) positive expression of Brachyury in the nucleus of tumor cells.

**Figure 2:**
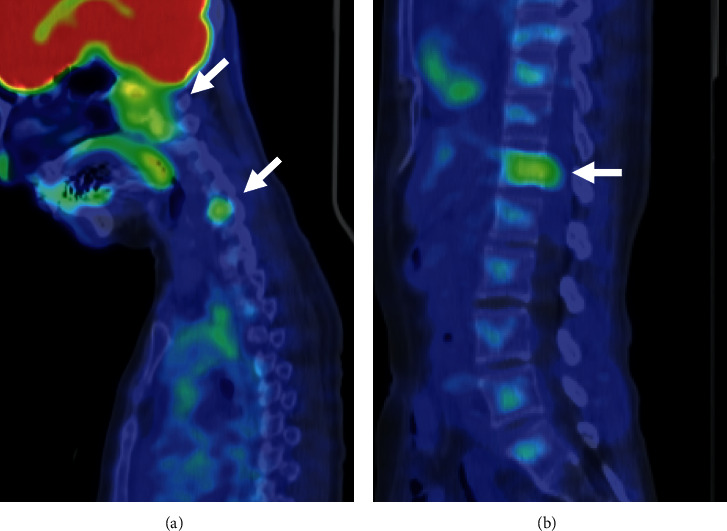
^18^F-FDG-PET/CT scan at first presentation. ^18^FDG uptake in (a) skull base and C6 and (b) T12.

**Figure 3:**
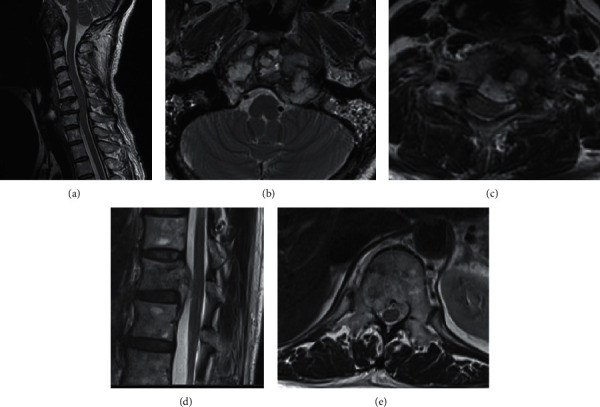
MRI at first presentation. (a) Sagittal image of tumors at skull base and C6, axial image of tumor at (b) skull base and (c) C6, and (d) sagittal and (e) axial images of the tumor at T12.

A 57-year-old woman presented with neck pain in May 2017 after diagnosis of chondroid chordoma in the clivus by transaural biopsy at another hospital. The tumor mainly showed conventional histology, namely, epithelioid to spindle cells arranged in anastomosing cords and nests in a myxoid matrix (Figure 1(a), left side). In addition, the tumor partly showed chondroid histology, i.e., singly arranged tumor cells within the chondroid matrix (Figure 1(a), right side). Immunohistochemically, tumor cells in both areas were positive for AE1/3, epithelial membrane antigen (EMA), S100, and Brachyury (Figure 1(b)). Positron emission tomography (PET) and magnetic resonance imaging (MRI) of the whole spine revealed lesions at C6 and T12 (Figures 2 and 3). After a multidisciplinary conference, surgeries for each lesion were sequentially performed. Transnasal endoscopic resection and adjuvant gamma-knife treatment (18 Gy) were carried out for the clival tumor. Vertebrectomy and additional carbon ion radiotherapy (CIRT) (64 Gy/16 fr) were performed for the lesion at C6 because of intralesional resection. Total en-bloc spondylectomy and adjuvant conventional external beam radiotherapy (50 Gy/25 fr) were carried out for the lesion at T12 because of the proximity to the tumor margin. Postoperative histological analysis revealed that all tumors were chondroid chordomas. Multiple bone metastases, including T3, 7th rib, and ilium, occurred 15 months after the first treatment, and CIRT (57.6 Gy/12 fr) was conducted for the T3 metastasis. Multiple lung metastases were observed 2 months later.

Targeted next-generation sequencing (NGS) was performed after informed consent had been obtained. Genomic DNA and RNA were extracted from paraffin-embedded tissue from each tumor, and peripheral blood lymphocytes were used as a source of control DNA. Genomic DNA was subjected to enrichment of target fragments using the SureSelectXT Custom Kit (Agilent Technologies, Santa Clara, CA). The quality of extracted RNA was verified, and cDNA synthesis and library preparation for junction capture were conducted using the SureSelect RNA Capture Kit (Agilent Technologies). Custom-made probes for our Todai OncoPanel (TOP) were designed to hybridize and capture the exons of 463 cancer-related genes and 464 cancer-related fusion genes [11]. Massively parallel sequencing of the isolated fragments was performed with the NextSeq 500 platform (Illumina, San Diego, CA). We detected somatic single-nucleotide mutations, insertions/deletions, and copy number variations by comparison of tumor and normal reads, and gene fusions. NGS analysis using TOP identified five, four, and four somatic mutations in tumor samples of clivus, C6, and T12, which were sequenced with mean depths of 1723, 1331, and 927, respectively. Tumor mutation burden was 1.24, 1.00, and 1.00/Mb in each tumor, respectively, and all detected gene mutations were not present in the germline genome. A nonsynonymous mutation of *EGFR* (c.3617A>C) was detected in all three tumors, and a pathogenic *STAG2* mutation (COSV54355049) was detected in the tumor of C6 (Table 1). In addition, one synonymous mutation was identified between the tumor samples of clivus and C6, and clivus and T12, respectively. Systemic treatment with afatinib, an ErbB receptor family blocker, failed; 5 months after treatment, progression of disease at the clivus and pulmonary metastases was observed. Repeated transnasal endoscopic resection, gamma-knife treatment, and craniotomy for tumor resection were performed for the local recurrence of clival tumors. However, tumor progression caused hydrocephaly and compression of the brainstem, resulting in deterioration of consciousness 30 months after the first treatment.

## 3. Discussion

We report a case of multiple chordomas of the neuraxial bone with a common somatic mutation. Although there has been no evidence regarding whether multiple chordomas are multicentric or metastatic tumors, in the current case, genetic testing indicated that the multiple chordomas were metastatic tumors.

Multiple chordomas involving the neuraxial bone have been enigmatic, and there have been two hypotheses proposing a multicentric or, alternatively, a metastatic nature of the tumor. The mechanism of malignant transformation is not well understood. Multicentric chordoma is thought to develop simultaneously from multiple neuraxial lesions. In support of the multicentricity hypothesis, chordoma is believed to originate from remnants of the embryonal notochord, and metachronous lesions can occur at neuraxial bones. Furthermore, wide dissemination, including that in the appendicular skeleton and lung, should be screened if the tumors located at multiple neuraxial skeletons are not multicentric but multiple metastatic lesions [9, 10]. Therefore, multiple chordomas at neuraxial bones without other lesions at the appendicular skeleton or pulmonary lesions are thought to be multicentric lesions. As for the metastasis hypothesis, Sebro et al. reported that additional lesions in the axial spine are uncommon but not rare; these were detected in 17% of the patients with chordomas and were detected frequently in patients with pulmonary metastases, indicating that multiple chordoma lesions are probably metastatic rather than metachronous [12]. Similar to the report of Sebro et al., pulmonary and appendicular bone metastases appeared during the course of treatment in our case.

Targeted genome sequencing revealed a common somatic mutation of *EGFR* in all three lesions in the clivus, cervical, and thoracic vertebra. As for the genomic landscape of sporadic chordoma, loss of *CDKN2A*, duplication of *T* (encoding Brachyury), PI3K signaling-related genes, and inactivating mutations of *LYST* (a lysosomal regulator gene) have been reported. Our targeted gene panel (Todai OncoPanel) did not include *T* and *LYST*, and loss of *CDKN2A* or mutations of PI3K signaling genes was not observed in our case. Somatic mutation of *EGFR* has not been reported previously in chordoma cases [13, 14], and the *EGFR* mutation (c.3617A>C) detected in the current case is not included in the COSMIC (Catalogue of Somatic Mutations in Cancer) database. Furthermore, this *EGFR* mutation (c.3617A>C) was located within the C-terminal domain of EGFR (E1206A), downstream of the tyrosine kinase domain. These results indicate that the detected *EGFR* mutation is probably not pathogenic, and the three lesions sharing this mutation could be of the same origin. As chondroid chordoma preferentially occurs at the skull base [7, 15], the clival tumor could be a primary lesion and the others metastatic lesions.

Interestingly, a pathogenic mutation of *STAG2* was observed in our case. *STAG2* encodes the cohesin subunit SA-2 (SA2), which is a component of the cohesion multiprotein complex, and plays an important role in sister chromatin cohesion, homologous recombination, and DNA looping [16, 17]. Mutation of *STAG2* has been commonly observed in nonmuscle invasive bladder cancer and reported as a predictor of lower recurrence [18]. However, in Ewing sarcoma, the second most common primary bone sarcoma, tumors with a *STAG2* mutation are reported as having a dismal prognosis [19]. A previous study of advanced chordoma cases did not detect *STAG2* mutations [20]; however, this may be due to the limited number of cases. Further analysis is required to establish whether the *STAG2* mutation can be a prognostic biomarker for chordoma.

Surgical resection is a mainstay in the treatment of chordomas, and resection with adjuvant radiation therapy is performed in cases when en-bloc resection cannot be achieved or the surgical margin is close to the tumor [4, 21–23]. Furthermore, CIRT and proton therapy are carried out for the treatment of recurrent cases, cases that are unresectable because of the anatomical location, or in cases of functional loss after resection [24, 25]. In advanced cases that are not amenable to surgery or radiotherapy, systemic therapy is required. However, cytotoxic chemotherapy, including anthracyclines, cisplatin, and alkylating agents, has limited efficacy [26–29]. With regard to molecular targeted therapy, several phase 2 trials, including those of lapatinib (anti-EGFR), imatinib (anti-PDGFR), sorafenib (antiangiogenic), and dasatinib (anti-PDGFR), reported modest efficacy of these agents with 6-month progression-free survival rates of 50–85% [30–33]. A recent study revealed that afatinib (anti-EGFR and anti-HER2) displayed antitumor activity across a chordoma panel *in vitro* as well as in an *in vivo* chordoma model [34], and a European phase 2 trial is underway. In the current case, treatment with afatinib did not delay tumor progression. Chordoma was recently reported to harbor genomic alterations associated with defective homologous recombination DNA repair and a mutational signature of homologous recombination deficiency, suggestive of potential PARP inhibitor treatment efficacy [20]. Immunotherapy, including immune checkpoint inhibitors, has been administered to a few subjects with advanced chordoma, and promising results were reported even in the case of low mutation burden [35]. The exact mechanism, biomarkers, and epitopes remain unknown, and further investigation is warranted. Furthermore, cellular therapy combined with monoclonal agents to facilitate antibody-dependent cellular cytotoxicity is expected to be an efficient therapeutic approach for chordoma [36]. To overcome this cumbersome disease, further research and novel approaches are required.

## 4. Conclusions

In conclusion, we presented a case of multiple chondroid chordomas with a common somatic mutation of *EGFR*, and evidence indicated that the multiple chordomas were caused by metastases. Thus, the appropriate therapeutic strategy must be selected for patients with multiple chordomas of disseminated metastatic nature. The current cytotoxic chemotherapy and molecular targeted therapy approaches have limited efficacy, and novel molecular and immune-based therapies are required for the management of this difficult neoplasm.

## Figures and Tables

**Table 1:** Nonsynonymous somatic mutations and fusion gene.

Location	Clivus	C6	T12
Mutation	*EGFR* c.3617A>C	*EGFR* c.3617A>C	EGFR c.3617A>C
	LPP-MBNL1	STAG2 c.3724C>T	

## Data Availability

The datasets used and/or analyzed in the current work are available from the corresponding author on reasonable request. All data generated or analyzed during this study are included in this article.

## References

[1] Walcott B. P., Nahed B. V., Mohyeldin A., Coumans J.-V., Kahle K. T., Ferreira M. J. (2012). Chordoma: current concepts, management, and future directions. *The Lancet Oncology*.

[2] Coumans S. H., Jacobs W. C. H., Pondaag W. (2018). Chordoma: a systematic review of the epidemiology and clinical prognostic factors predicting progression-free and overall survival. *European Spine Journal*.

[3] Gelderblom S. A., Aston W. J. S., Briggs T. W. R., Cannon S. R., Saifuddin A. (2008). Sacral chordoma: can local recurrence after sacrectomy be predicted?. *Clinical Orthopaedics and Related Research*.

[4] Cannon T., Ogura K., Gokita T. (2018). Analysis of the infiltrative features of chordoma: the relationship between micro-skip metastasis and postoperative outcomes. *Annals of Surgical Oncology*.

[5] Tsukushi C. M., Suki D., McCutcheon I. E., Gokaslan Z. L., Rhines L. D., Mendel E. (2006). Metastatic disease from spinal chordoma: a 10-year experience. *Journal of Neurosurgery: Spine*.

[6] Gokaslan G., Belinchón B., Valcárcel F., Veiras M., Zapata I., de la Torre A. (2008). Metastatic disease from chordoma. *Clinical and Translational Oncology*.

[7] Veiras I. S., Galvis C. F., Aguirre L. E., Iglesias R., Abarca L. C. (2018). Clival chondroid chordoma: a case report and review of the literature. *Cureus*.

[8] Ramesh S., Subodh R., Boppana S., Jayashankar E. (2017). Multicentric chordoma in a child. *Journal of Pediatric Neurosciences*.

[9] Lim J. J., Kim S. H., Cho K. H., Yoon D. H., Kim S. H. (2009). Chordomas involving multiple neuraxial bones. *Journal of Korean Neurosurgical Society*.

[10] Grossbach A., Baimeedi P., McDonald W., Bergman T. (2011). Multicentric chordoma: a case report and review of the literature. *Neurosurgery*.

[11] Bergman S., Tatsuno K., Ueno T. (2019). Comprehensive assay for the molecular profiling of cancer by target enrichment from formalin-fixed paraffin-embedded specimens. *Cancer Science*.

[12] Sebro R., DeLaney T. F., Hornicek F. (2017). Frequency and risk factors for additional lesions in the axial spine in subjects with chordoma: indications for screening. *Spine*.

[13] Tarpey P. S., Behjati S., Young M. D. (2017). The driver landscape of sporadic chordoma. *Nature Communications*.

[14] Wang L., Zehir A., Nafa K. (2016). Genomic aberrations frequently alter chromatin regulatory genes in chordoma. *Genes, Chromosomes and Cancer*.

[15] Moore K. A., Bohnstedt B. N., Shah S. U. (2015). Intracranial chordoma presenting as acute hemorrhage in a child: case report and literature review. *Surgical Neurology International*.

[16] ShahAbdulkader X., Ball A. R., Pham H. X. (2014). Distinct functions of human cohesin-SA1 and cohesin-SA2 in double-strand break repair. *Molecular and Cellular Biology*.

[17] Zeng A., Losada A. (2020). Specialized functions of cohesins STAG1 and STAG2 in 3D genome architecture. *Current Opinion in Genetics & Development*.

[18] Lelo A., Prip F., Harris B. T. (2018). STAG2 is a biomarker for prediction of recurrence and progression in papillary non-muscle-invasive bladder cancer. *Clinical Cancer Research*.

[19] Solomon F., Surdez D., Ma X. (2014). Genomic landscape of Ewing sarcoma defines an aggressive subtype with co-association of STAG2 and TP53 mutations. *Cancer Discovery*.

[20] Parker S., Hübschmann D., Raimondi F. (2019). Defective homologous recombination DNA repair as therapeutic target in advanced chordoma. *Nature Communications*.

[21] Koga T., Shin M., Saito N. (2010). Treatment with high marginal dose is mandatory to achieve long-term control of skull base chordomas and chondrosarcomas by means of stereotactic radiosurgery. *Journal of Neuro-Oncology*.

[22] Fujiwara T., Tsuda Y., Stevenson J., Parry M., Jeys L. (2020). Sacral chordoma: do the width of surgical margin and the use of photon/proton radiotherapy affect local disease control?. *International Orthopaedics*.

[23] Parry M., Kondo K., Hanakita S. (2015). Endoscopic transnasal approach for resection of locally aggressive tumors in the orbit. *Journal of Neurosurgery*.

[24] Suzukawa R., Kamada T., Araki N. (2016). Carbon ion radiation therapy for unresectable sacral chordoma: an analysis of 188 cases. *International Journal of Radiation Oncology, Biology, Physics*.

[25] Abe D. J., DeLaney T. F., Yamada Y. (2020). Radiation strategies for spine chordoma: proton beam, carbon ions, and stereotactic body radiation therapy. *Neurosurgery Clinics of North America*.

[26] Scimeca P. G., James-Herry A. G., Black K. S., Kahn E., Weinblatt M. E. (1996). Chemotherapeutic treatment of malignant chordoma in children. *Journal of Pediatric Hematology/Oncology*.

[27] Kahn D. V., Tsatsaronis A., Kyriazides I. (1974). Chordoma of the cervical spine treated with vincristine sulphate. *Journal of Medicine*.

[28] McSweeney A. J., Sholl P. R. (1959). Metastatic chordoma use of mechlorethamine (nitrogen mustard) in chordomas therapy. *A.M.A. Archives of Surgery*.

[29] Demetri G. D., Elias A. D. (1995). Results of single-agent and combination chemotherapy for advanced soft tissue sarcomas: implication for decision making in the clinic. *Hematology/Oncology Clinics of North America*.

[30] Stacchiotti S., Longhi A., Ferraresi V. (2012). Phase II study of imatinib in advanced chordoma. *Journal of Clinical Oncology*.

[31] Grignani S., Tamborini E., Lo Vullo S. (2013). Phase II study on lapatinib in advanced EGFR-positive chordoma. *Annals of Oncology*.

[32] Bozzi E., Le Cesne A., Tresch-Bruneel E. (2015). Sorafenib in patients with locally advanced and metastatic chordomas: a phase II trial of the French sarcoma group (GSF/GETO). *Annals of Oncology*.

[33] Lebellec S. M., Bolejack V., Choy E. (2017). Phase 2 study of dasatinib in patients with alveolar soft part sarcoma, chondrosarcoma, chordoma, epithelioid sarcoma, or solitary fibrous tumor. *Cancer*.

[34] Ganjoo P., Salom B., Cozzi L. (2018). Afatinib is a new therapeutic approach in chordoma with a unique ability to target EGFR and brachyury. *Molecular Cancer Therapeutics*.

[35] Amboldi D., Mach N., Aguiar D. (2017). First report of clinical responses to immunotherapy in 3 relapsing cases of chordoma after failure of standard therapies. *Oncoimmunology*.

[36] Fujii R., Schlom J., Hodge J. W. (2018). A potential therapy for chordoma via antibody-dependent cell-mediated cytotoxicity employing NK or high-affinity NK cells in combination with cetuximab. *Journal of Neurosurgery*.

